# Supplementary material for: Influence of Cluster-Situated Regulator PteF in Filipin Biosynthetic Cluster on Avermectin Biosynthesis in Streptomyces avermitilis
Source: Biology (Basel). 2024 May 15;13(5):344. doi: 10.3390/biology13050344 (PMC11118972; doi:10.3390/biology13050344)
Supplement: Supplementary file 1 [file biology-13-00344-s001.zip › biology-3003482-supplementary.pdf]

Supplementary Materials

Influence of Cluster-Situated Regulator PteF in Filipin Biosynthetic Cluster on Avermectin Biosynthesis in *Streptomyces Avermitilis*

Guozhong Du [1], Xue Yang [1], Zhengxiong Wu [1], Minghui Pan [1,2], Zhuoxu Dong [1,2], Yanyan Zhang [1], Wensheng Xiang [1,2,*], Shanshan Li[1,*]

[1] State Key Laboratory for Biology of Plant Diseases and Insect Pests, Institute of Plant Protection, Chinese Academy of Agricultural Sciences, No. 2 Yuanmingyuan West Road, Haidian District, Beijing 100193, China; dgz1005@163.com (G.D.); 82101211024@caas.cn (X.Y.); 17513232046@163.com (Z.W.); mhlu@163.com (M.P.); d15945317995@163.com (Z.D.); yyzhang@ippcaas.cn (Y.Z.)

[2] School of Life Science, Northeast Agricultural University, No. 59 Mucai Street, Xiangfang District, Harbin 150030, China

* Correspondence: xiangwensheng@neau.edu.cn (W.X.); lishanshan01@caas.cn (S.L.)

Table S1. Strains and plasmids used in this work

Name	Description	Source
Strains		
Escherichia coli		
JM109	General cloning host for plasmid manipulation	Novagen
ET12567(pUZ8002)	Donor strain for conjugation between *E. coli* and *Streptomyces*	[1]
Streptomyces avermitilis		
S0	Low-yielding strain of avermectins, derived from *S. avermitilis* MA-4680 by random mutagenesis using ultraviolet mutagenesis and *N*-methyl-*N'*-nitroso-*N*-nitrosoguanidine (NTG) mutagenesis	CGMCC 4.8011
savΔ*pteF*	In-frame deleting the *pteF* gene in S0	This work
savΔ*pteR*	In-frame deleting the *pteR* gene in S0	This work
Plasmids		
pKC1139	Am^r, integrative *E. coli*-*Streptomyces* shuttle vector for gene disruption	[2]
pKC1139::Δ*pteF*	For in-frame deleting the *pteF* gene, generated from pKC1139	This work
pKC1139::Δ*pteR*	For in-frame deleting the *pteR* gene, generated from pKC1139	This work

Table S2. Primers used in this work

Primers	Sequence (5'-3') [1]	Usage
ΔpteR-LF	**TAAAACGACGGCCAGTGCCAAGCTT**ACCACGTGCGCTGATCAC	For amplification of the left homologous arm
ΔpteR-LR	TGGATCCGTGCTCTGCCTC	of *pteR*
ΔpteR-RF	**ACTGACGAGGCAGAGCACGGATCCA**GGCGAACGGCGCCTTCCAA	For amplification of the right homologous
ΔpteR-RR	**CGCGCGCGGCCGCGGATCCTCTAGA**GGCAGCACCTCCTCGAAGT	arm of *pteR*
ΔpteF-LF	**TAAAACGACGGCCAGTGCCAAGCTT**CCTGGAACCAGTCGGAGGC	For amplification of the left homologous arm
ΔpteF-LR	ACACAGGCCCGGAGTTGCA	of *pteF*
ΔpteF-RF	**GGCACCTGCAACTCCGGGCCTGTGT**GTCGAGCACAGGATCGCCG	For amplification of the left homologous arm
ΔpteF-RR	**CGCGCGCGGCCGCGGATCCTCTAGA**TGATGACGCGCTCGATGTT	of *pteF*
ΔpteRY-F	GAAGGAACGGAAGGAAGGGAG	For short fragment verification of *pteR*
ΔpteRY-R	CAGGAAACTTGGGCGAAATAAA	deletion mutant
ΔpteFY-F	TCGGAGGTAAAGGCATGAACG	For short fragment verification of *pteF*
ΔpteFY-R	AGATGAAAGATGAATCGGAGGG	deletion mutant
ΔpteRLY-F	CAAGCCCGTCGAGGAACTG	For long fragment verification of *pteR*
ΔpteRLY-R	CCGTGGTTGTTGCCGTAGAG	deletion mutant
ΔpteFLY-F	TGAGCAGGTCAAGGGAGGC	For long fragment verification of *pteF*
ΔpteFLY-R	GAGTCGTTCCTTTACGGTTTCG	deletion mutant

[1] Bold type characters indicate the homologous fragments

Table S3. Genes significantly differentially expressed in PKS BGCs

Cluster	Production	Gene id	Genome description	$\log_2$ (fold change) at day 2[1]	$\log_2$ (fold change) at day 6[1]
pks11	Unknown	*GM000247*	putative partial polyketide synthase (type-II KS), partial	**-1.04**	1.00
		GM000249	putative polyketide synthase (type-II AT + aminotransferase), partial	0.22	**-1.00**
pte	Filipin	*GM000690*	oleoyl-ACP hydrolase	**2.37**	**2.88**
		GM000691	cholesterol oxidase	**2.42**	**2.76**
		GM000695	ferredoxine	**-2.24**	**-2.33**
		GM000696	cytochrome P450 hydroxylase	**-1.98**	**-2.55**
		GM000697	cytochrome P450 hydroxylase	**-1.85**	**-2.29**
		GM000698	cytochrome P450	**-1.72**	**-2.39**
		GM000699	crotonyl-CoA reductase	**-1.96**	**-2.44**
		GM000700	modular polyketide synthase, partial	**-1.78**	**-2.25**
		GM000701	modular polyketide synthase, partial	**-1.84**	**-2.20**
		GM000702	modular polyketide synthase, partial	**-1.93**	**-2.20**
		GM000703	modular polyketide synthase, partial	**-1.82**	**-2.19**
		GM000704	modular polyketide synthase, partial	**-1.78**	**-2.28**
		GM000705	modular polyketide synthase, partial	**-1.91**	**-2.29**
		GM000706	modular polyketide synthase, partial	**-2.04**	**-2.14**
		GM000707	modular polyketide synthase, partial	**-2.13**	**-2.09**
		GM000708	modular polyketide synthase, partial	**-2.01**	**-2.14**
		GM000709	modular polyketide synthase, partial	**-2.07**	**-2.12**
		GM000710	modular polyketide synthase, partial	**-2.53**	**-2.27**
		GM000711	modular polyketide synthase, partial	**-2.00**	**-2.19**

		GM000712	modular polyketide synthase, partial	**-2.16**	**-1.74**
		GM000713	modular polyketide synthase, partial	**-1.87**	**-2.22**
		GM000714	modular polyketide synthase, partial	**-1.94**	**-2.22**
		GM000715	modular polyketide synthase, partial	**-2.16**	**-2.25**
		GM000716	modular polyketide synthase, partial	**-1.99**	**-2.26**
		GM000717	modular polyketide synthase, partial	**-1.88**	**-2.14**
		GM000718	modular polyketide synthase, partial	**-1.94**	**-2.23**
		GM000719	modular polyketide synthase, partial	**-3.23**	**-1.77**
		GM000720	modular polyketide synthase, partial	**-1.89**	**-2.17**
		GM000721	modular polyketide synthase, partial	**-1.70**	**-2.35**
ave	Avermectin	*GM000868*	type I polyketide synthase AVES 1, partial	**1.56**	-0.04
		GM000881	type I polyketide synthase AVES 2, partial	**-1.58**	**1.74**
		GM000884	type I polyketide synthase AVES 2, partial	**-1.72**	0.87
		GM000893	type I polyketide synthase AVES 4, partial	**-1.03**	0.14
		GM000897	type I polyketide synthase AVES 4, partial	**-1.24**	-0.02
pks3	Unknown	*GM002527*	putative 3-oxoacyl-ACP synthase III	-0.99	**1.01**
		GM002532	putative acyl-CoA synthetase, partial	**-4.58**	-0.06
		GM002533	putative modular polyketide synthase, partial	**-1.15**	0.47
		GM002534	putative modular polyketide synthase, partial	**-2.07**	0.68
		GM002535	putative modular polyketide synthase, partial	**-2.88**	0.39
		GM002536	putative modular polyketide synthase, partial	**-4.04**	-0.18
		GM002537	putative acyl carrier protein	**-1.65**	-0.11
pks5	Unknown	*GM002641*	putative modular polyketide synthase, partial	0.37	**1.45**
		GM002643	putative modular polyketide synthase, partial	**-1.79**	-0.10
		GM002644	putative regulatory protein, partial	0.52	**1.97**
		GM002646	putative regulatory protein, partial	**1.21**	-0.60

pks9	Unknown	*GM002652*	putative 3-oxoacyl-ACP synthase II (probably chain length factor)	**-1.35**	0.24
		GM002654	putative cytochrome P450	-0.24	**-1.42**
		GM002657	putative ABC transporter permease protein, partial	**-1.03**	**-2.94**
		GM002658	putative ABC transporter permease protein, partial	-0.63	**2.70**
pks8	Unknown	*GM004430*	putative acyl carrier protein, partial	**1.38**	**-1.25**
		GM004434	putative 3-oxoacyl-ACP synthase I, partial	--	**1.43**
		GM004440	putative acyl carrier protein	**-1.05**	0.55
olm	Oligomycin	*GM005321*	putative P450-like protein	**1.25**	0.41
		GM005323	modular polyketide synthase, partial	**-1.22**	0.36
		GM005326	modular polyketide synthase, partial	0.86	**1.06**
		GM005329	modular polyketide synthase, partial	**-2.82**	0.48
pks4	Unknown	*GM008451*	putative modular polyketide synthase, partial	**-1.60**	0.21
		GM008452	putative modular polyketide synthase, partial	**-1.55**	0.78
		GM008456	putative UDP-glucose: sterol glucosyltransferase, partial	0.43	**2.43**
		GM008457	cytochrome P450 hydroxylase	**-1.48**	**1.59**

[1] Bold type characters indicate the changing was significant.

Table S4. Genes significantly differentially expressed at day 2

Gene id	Genome description	log_2 (fold change)
GM000066	putative ATP/GTP-binding protein	-4.76
GM000095	conserved hypothetical protein	-2.12
GM000202	\	-5.17
GM000222	putative TetR-family transcriptional regulator	-1.68
GM000273	putative IS5 family IS1647-like transposase	1.10
GM000279	hypothetical protein	-1.12
GM000281	helicase	-4.66
GM000361	F420-dependent oxidoreductase	-2.15
GM000375	\	-3.76
GM000499	hypothetical protein, partial	-3.80
GM000630	putative IS110 family ISLxx2-like transposase	-2.61
GM000631	\	-5.51
GM000690	oleoyl-ACP hydrolase	2.37
GM000691	cholesterol oxidase	2.42
GM000692	LuxR family transcriptional regulator	-1.38
GM000695	ferredoxine	-2.24
GM000696	cytochrome P450 hydroxylase	-1.98
GM000697	\	-1.85
GM000698	cytochrome P450 hydroxylase	-1.72
GM000699	crotonyl-CoA reductase	-1.96
GM000700	hypothetical protein	-1.78
GM000701	Acyl transferase	-1.84
GM000702	polyketide synthase, partial	-1.93
GM000703	polyketide synthase	-1.82

GM000704	modular polyketide synthase	-1.78
GM000705	modular polyketide synthase	-1.91
GM000706	modular polyketide synthase	-2.04
GM000707	hypothetical protein, partial	-2.13
GM000708	\	-2.01
GM000709	polyketide synthase	-2.07
GM000710	polyketide synthase	-2.53
GM000711	hypothetical protein, partial	-2.00
GM000712	\	-2.16
GM000713	\	-1.87
GM000714	polyketide synthase, partial	-1.94
GM000715	Malonyl CoA-acyl carrier protein transacylase	-2.16
GM000716	hypothetical protein, partial	-1.99
GM000717	hypothetical protein, partial	-1.88
GM000718	modular polyketide synthase	-1.94
GM000719	hypothetical protein, partial	-3.23
GM000720	modular polyketide synthase	-1.89
GM000721	EbeA	-1.70
GM000722	\	-3.42
GM000723	putative Tn3 family ISXc5-like transposase	-2.00
GM000749	\	-1.27
GM000760	hypothetical protein	-4.04
GM000773	\	-3.97
GM000932	putative LacI-family transcriptional regulator	-1.24
GM000983	lycopene cyclase	1.23
GM001009	putative membrane protein	-1.85

GM001042	hypothetical protein	-2.85
GM001103	hypothetical protein	2.34
GM001184	ABC transporter substrate-binding protein	-1.24
GM001185	putative branched-chain amino acid ABC transporter permease protein	-1.06
GM001186	putative branched-chain amino acid ABC transporter permease protein	-1.08
GM001187	putative branched-chain amino acid ABC transporter ATP-binding protein	-1.79
GM001189	putative RNA polymerase ECF-subfamily sigma factor	-1.30
GM001201	alkanesulfonate monooxygenase	-3.71
GM001272	hypothetical protein	-1.43
GM001503	putative alpha-glucuronidase	-1.17
GM001685	hypothetical protein	-1.42
GM001689	3-ketosteroid-delta-1-dehydrogenase	-2.51
GM001879	putative secreted chitinase I	-1.37
GM001883	ArsR family transcriptional regulator	-1.00
GM001915	putative 3-(2-hydroxyphenyl) propionic acid transporter	-1.92
GM001933	hypothetical protei	-1.01
GM002037	\	-5.38
GM002248	putative trypsin-like protease, secreted	-1.47
GM002288	putative xanthine dehydrogenase	-1.33
GM002328	putative hydrolase	1.21
GM002367	hypothetical protein	-1.26
GM002424	\	-4.50
GM002484	hypothetical protein	-1.98
GM002487	putative transcriptional regulator	-1.75
GM002499	alpha-mannosidase	-1.05
GM002500	alpha-mannosidase	-1.07

GM002532	\	-4.58
GM002544	\	-4.93
GM002590	\	-4.81
GM002592	\	-1.39
GM002846	\	-4.89
GM002907	putative multiple sugar ABC transporter permease protein	-1.73
GM002934	putative membrane protein	1.02
GM003206	putative transmembrane sulfate transport protein	-1.23
GM003291	50S ribosomal protein L33 1	-1.08
GM003407	amino acid permease	-1.33
GM003410	putative ABC transporter permease protein	-1.15
GM003414	regulatory protein	-5.22
GM003473	\	-3.02
GM003574	putative regulatory protein	-1.51
GM003575	hypothetical protein	-1.22
GM003618	AsnC family transcriptional regulator	-1.47
GM003782	hypothetical protein	-5.50
GM003888	hypothetical protein	-2.59
GM003939	putative regulatory protein	2.19
GM003940	hypothetical protein	1.79
GM003976	hypothetical protein	3.98
GM004377	hypothetical protein	-3.38
GM004378	putative ATP-binding protein	-4.49
GM004508	peroxidase	-3.07
GM004833	putative ABC transporter permease protein	-3.74
GM004874	hypothetical protein	-1.18

GM005013	putative export protein	-1.01
GM005014	hypothetical protein	-1.15
GM005091	putative peptide ABC transporter solute-binding protein	-1.35
GM005092	putative peptide ABC transporter permease protein	-1.93
GM005270	integrase, partial	-5.48
GM005285	\	-3.00
GM005289	hypothetical protein	-2.02
GM005356	putative two-component system sensor kinase	-1.06
GM005357	membrane protein	-1.80
GM005456	putative neutral zinc metalloprotease, secreted	-1.26
GM005488	putative peptide ABC transporter ATP-binding protein	1.66
GM005812	\	-3.74
GM005847	hypothetical protein	1.02
GM005862	putative nitrate extrusion protein	-1.08
GM006066	putative secreted ribonuclease	-1.13
GM006289	hypothetical protein	-1.42
GM006340	hypothetical protein	-2.84
GM006412	putative IclR-family transcriptional regulator	-2.29
GM006521	putative hydrolase	-2.77
GM006751	Putative ABC transporter ATP-binding protein SAV_5847	1.37
GM006791	putative integral membrane protein	2.49
GM007052	putative RNA polymerase ECF-subfamily sigma factor	-5.05
GM007190	putative membrane protein	-3.20
GM007217	putative two-component system response regulator	1.57
GM007707	putative MerR-family transcriptional regulator	1.02
GM007923	\	1.95

GM008086	\	-1.08
GM008306	putative ABC transporter ATP-binding protein	-5.17
GM008344	Urea amidohydrolase subunit gamma	-1.24
GM008348	Urea amidohydrolase beta gamma	-1.35
GM008357	\	-1.23
GM008417	putative aminotransferase	-4.83
GM008507	hypothetical protein	-1.32
GM008693	protein phosphatase	-3.13
GM008796	amino acid ABC transporter amino acid-binding protein	-4.44
GM008850	dehydrogenase	-2.77
GM008864	hypothetical protein	-1.26
GM009061	tail protein, partial	-4.55
GM009109	aromatic compound degradation protein PaaI	-1.30

Table S5. Genes significantly differentially expressed at day 6

Gene id	Genome description	log_2 (fold change)
GM000121	\	1.22
GM000177	putative IS5 family ISJp4-like transposase	1.39
GM000335	hypothetical protein	-1.08
GM000689	NUDIX hydrolase	2.40
GM000690	oleoyl-ACP hydrolase	2.88
GM000691	cholesterol oxidase	2.76
GM000692	LuxR family transcriptional regulator	-1.50
GM000695	ferredoxine	-2.33
GM000696	cytochrome P450 hydroxylase	-2.55
GM000697	\	-2.29
GM000698	cytochrome P450 hydroxylase	-2.39
GM000699	crotonyl-CoA reductase	-2.44
GM000700	hypothetical protein	-2.25
GM000701	Acyl transferase	-2.20
GM000702	polyketide synthase, partial	-2.20
GM000703	polyketide synthase	-2.19
GM000704	modular polyketide synthase	-2.28
GM000705	modular polyketide synthase	-2.29
GM000706	modular polyketide synthase	-2.14
GM000707	hypothetical protein, partial	-2.09
GM000708	\	-2.14
GM000709	polyketide synthase	-2.12
GM000710	polyketide synthase	-2.27
GM000711	hypothetical protein, partial	-2.19

GM000712	\	-1.74
GM000713	\	-2.22
GM000714	polyketide synthase, partial	-2.22
GM000715	Malonyl CoA-acyl carrier protein transacylase	-2.25
GM000716	hypothetical protein, partial	-2.26
GM000717	hypothetical protein, partial	-2.14
GM000718	modular polyketide synthase	-2.23
GM000720	modular polyketide synthase	-2.17
GM000721	modular polyketide synthase	-2.35
GM000723	putative Tn3 family ISXc5-like transposase	-2.06
GM001082	hypothetical protein	1.05
GM001103	hypothetical protein	1.18
GM001109	putative neutral zinc metalloprotease, secreted	-1.20
GM001111	\	-1.36
GM001271	hypothetical protein	-2.09
GM001272	hypothetical protein	-2.21
GM001418	monooxygenase, flavin-binding family	-1.68
GM001420	hypothetical protein	-1.62
GM001452	lipoprotein	1.05
GM001513	acetyl xylan esterase	1.32
GM001997	formate dehydrogenase, major subunit	1.12
GM002248	putative trypsin-like protease, secreted	-1.50
GM002371	putative integral membrane protein	-1.34
GM002372	putative integral membrane protein	-1.12
GM002580	inorganic polyphosphate/ATP-NAD kinase	1.17
GM002597	putative high-affinity nickel-transport protein	1.57

GM002996	putative transmembrane efflux protein	-1.48
GM002997	TetR family transcriptional regulator	-1.01
GM003180	hypothetical protein	1.55
GM003209	peptidase	-1.26
GM003307	hypothetical protein	1.95
GM003532	hypothetical protein	1.05
GM003574	putative regulatory protein	-1.13
GM003575	hypothetical protein	-1.04
GM003685	hypothetical protein	1.10
GM003877	hypothetical protein	1.32
GM003939	putative regulatory protein	4.17
GM003940	hypothetical protein	3.69
GM003975	putative ABC transporter permease protein	1.54
GM003976	hypothetical protein	4.92
GM005007	hypothetical protein	-1.50
GM005008	hypothetical protein	-1.84
GM005009	Diaminopimelate epimerase	-1.48
GM005010	aminotransferase	-1.25
GM005011	peptide synthetase	-1.08
GM005092	putative peptide ABC transporter permease protein	4.31
GM005162	hypothetical protein	1.02
GM005256	\	1.75
GM005357	membrane protein	-1.53
GM005457	\	-1.36
GM005458	hypothetical protein	-1.16
GM005488	putative peptide ABC transporter ATP-binding protein	1.56

GM005579	hypothetical protein	-1.56
GM005992	putative polar amino acid ABC transporter substrate-binding protein	-1.34
GM006059	putative sodium: solute symporter	-1.18
GM006079	hypothetical protein	-1.34
GM006471	hypothetical protein	1.38
GM006472	putative DNA recombination and repair protein	1.59
GM006659	hypothetical protein	1.19
GM006727	\	1.81
GM006752	cobalt ABC transporter permease	2.08
GM006923	putative secreted alpha-amylase	1.05
GM007003	membrane protein	1.22
GM007004	collagen triple helix repeat family protein	1.07
GM007061	hypothetical protein	-1.08
GM007181	\	-1.74
GM007268	hypothetical protein	-1.24
GM007585	putative DNA hydrolase	1.83
GM007586	putative ADP-ribosylglycohydrolase	1.68
GM007587	nucleotidyltransferase	1.08
GM007910	putative oxidoreductase	-1.01
GM008263	membrane protein	1.20
GM008359	LytR family transcriptional regulator	1.23
GM008686	hypothetical protein	-1.26
GM008745	\	-1.27
GM009092	6-phosphogluconate dehydrogenase	-1.33

Table S6. Genes enriched in metabolic pathways with significant changes after *pteF* deletion

Metabolic pathway	Gene id		Gene name	Genome description	log_2 (fold change)	
	S0	MA-4680			day 2	day 6
**Day 2**						
**ABC transporters**	*GM001184*	*SAVERM_1190*	*livK1*	ABC transporter substrate-binding protein	-1.24	-0.78
	GM001185	*SAVERM_1191*	*livH1*	putative branched-chain amino acid ABC transporter permease protein	-1.06	-0.78
	GM001186	*SAVERM_1192*	*livM1*	putative branched-chain amino acid ABC transporter permease protein	-1.08	-0.67
	GM001187	*SAVERM_1193*	*livG1*	putative branched-chain amino acid ABC transporter ATP-binding protein	-1.79	-0.78
	GM002415	*SAVERM_2178*	*opuBC1*	glycine/betaine ABC transporter substrate-binding protein	-0.66	-0.40
	GM002424	*SAVERM_2184*	\	putative ABC transporter permease	-4.50	-0.51
	GM002494	*SAVERM_2247*	*xylF*	ABC transporter substrate-binding protein	-0.99	-0.30
	GM002498	*SAVERM_2251*	*ngcE*	ABC transporter substrate-binding protein	-0.50	0.01
	GM002907	*SAVERM_4980*	*msmF*	putative multiple sugar ABC transporter permease protein	-1.73	-0.16
	GM006477	*SAVERM_5632*	*mntC*	putative zinc/manganese transport system ABC transporter permease	-0.54	0.04
	GM006864	*SAVERM_5940*	*oleC5*	putative ABC transporter permease protein	0.91	-0.43
	GM006865	*SAVERM_5941*	*oleC4*	putative ABC transporter ATP-binding protein	0.95	-0.45
	GM008796	*SAVERM_7408*	\	amino acid ABC transporter amino acid-binding protein	-4.44	-0.02
**Sulfur metabolism**						
	GM002592	*SAVERM_2330*	*narB*	putative assimilatory nitrate reductase large subunit	-1.39	-0.28

	GM001201	*SAVERM_1208*		alkanesulfonate monooxygenase	-3.71	0.14
**Nitrogen metabolism**						
	GM006513	*SAVERM_5661*	*nirB*	putative nitrite reductase (NAD(P)H) large subunit	-0.87	-0.33
	GM005862	*SAVERM_5119*	*narK*	putative nitrate extrusion protein	-1.08	-0.18
	GM006947	*SAVERM_5997*	*glnA2*	putative glutamine synthetase	-0.56	-0.40
**Propanoate metabolism**						
	GM002244	*SAVERM_2031*	*acsA4*	putative acetyl-CoA synthetase	0.62	0.85
	GM008417	*SAVERM_7160*	\	putative aminotransferase	-4.83	0.86
	GM006049	*SAVERM_5277*	*accA1*	putative acetyl/propionyl CoA carboxylase alpha subunit	0.58	0.03
**Day 6 Glyoxylate and dicarboxylate metabolism**						
	GM002244	*SAVERM_2031*	*acsA4*	putative acetyl-CoA synthetase	0.62	0.85
	GM000699	*SAVERM_414*	*pteB*	crotonyl-CoA reductase	-1.96	-2.44
	GM002508	*SAVERM_2258*	*acnA*	aconitate hydratase	-0.29	-0.35
	GM000609	*SAVERM_348*	*katB*	putative catalase	0.07	-0.51
	GM006881	*SAVERM_5954*	*glnA1*	\	-0.03	-0.40
	GM006947	*SAVERM_5997*	*glnA2*	putative glutamine synthetase	-0.56	-0.40
**Two-component system**						
	GM008744	*SAVERM_7366*	*hydA*	putative cytochrome C3-like [NiFe] hydrogenase small subunit	0.06	-0.74

	GM007121	*SAVERM_6139*	*phoD1*	putative alkaline phosphatase	-0.30	-0.56
	GM008746	*SAVERM_7367*	*hydB*	putative cytochrome C3-like [NiFe] hydrogenase large subunit	-0.07	-0.60
	GM006947	*SAVERM_5997*	*glnA2*	putative glutamine synthetase	-0.56	-0.40
	GM006881	*SAVERM_5954*	*glnA1*		-0.03	-0.40
**Biosynthesis of various antibiotics**						
	GM000696	*SAVERM_412*	*pteD*	cytochrome P450 hydroxylase	-1.98	-2.55
	GM008419	*SAVERM_7162*	*cysK3*	putative cysteine synthase	-0.34	-0.67
**D-Amino acid metabolism**						
	GM005009	*SAVERM_3161*	*dapF2*	Diaminopimelate epimerase	-0.93	-1.48
	GM006060	*SAVERM_5285*	*speB*	putative agmatinase	0.34	-0.63
	GM008682	*SAVERM_7323*	*avsD*	putative diaminopimelate decarboxylase	0.37	0.97
**Glycerolipid metabolism**						
	GM007693	*SAVERM_6595*	\	putative aldehyde dehydrogenase	0.03	-0.50
	GM007927	*SAVERM_6783*	*plsC4*	putative 1-acylglycerol-3-phosphate O-acyltransferase	-0.09	-0.42
	GM008560	*SAVERM_7256*	*tgs*	\	-0.42	-0.64
**Propanoate metabolism**						
	GM006049	*SAVERM_5277*	*accA1*	putative acetyl/propionyl CoA carboxylase alpha subunit	0.58	0.03
	GM002244	*SAVERM_2031*	*acsA4*	putative acetyl-CoA synthetase	0.62	0.85

	GM004760	*SAVERM_3382*	*mcmA 2*	putative methylmalonyl-CoA mutase, alpha subunit	0.33	-0.47
	GM003600	*SAVERM_4376*	*bkdF*	putative branched-chain alpha keto acid dehydrogenase E1 alpha subunit	0.08	-0.36

References

1. Kieser, T.; Bibb, M.J.; Buttner, M.J.; Chater, K.F.; Hopwood, D.A. Practical *Streptomyces* genetics. *John Innes Found. Norwich.* **2000**; Volume *291*.
2. Bierman, M.; Logan, R.; O'Brien, K.; Seno, E.T.; Rao, R.N.; Schoner, B.E. Plasmid cloning vectors for the conjugal transfer of DNA from *Escherichia coli* to *Streptomyces* spp. *Gene*. **1992**, *116*, 43–49.
